# Development of Palliative and End of Life Care: The Current Situation in Saudi Arabia

**DOI:** 10.7759/cureus.4319

**Published:** 2019-03-26

**Authors:** Sami Alshammaray, Balaji Duraisamy, Yousef Albalawi, Savithiri Ratnapalan

**Affiliations:** 1 Palliative Care, King Fahd Medical City, Riyadh, SAU; 2 Palliative Care, Vision Realization Office, Ministry of Health, Riyadh, SAU; 3 Pediatrics, University of Toronto's Continuing Professional Development Office, SickKids Hospital, Toronto, CAN

**Keywords:** palliative care, last phase, care design group, pathway, vision 2030

## Abstract

As part of the health care reforms and transformation project of the Vision 2030, a group of expert healthcare professionals was tasked with the development of a model of care for patients with life-shortening illnesses in Saudi Arabia. This Care Design Group (CDG 1-3) held a series of workshops and conducted surveys and online discussions to systematically document and develop a model of care. These interventions were aimed at achieving a national standard of care. This short article is a description of this very successful process of development.

## Introduction

Palliative care in Saudi Arabia is still in its nascent stages. Even after two decades, palliative care is not widely available across Saudi Arabia. Meanwhile, the Ministry of Health (MOH) launched the Last Phase Initiative as part of the Transformation of Healthcare - Vision 2030. By 2030, the cancer burden in Saudi Arabia is expected to grow by five to 10-fold. This result is predicted by the changing demographics, with the age group most affected by cancer being the middle-aged and elderly [1]. The palliative care community in Saudi Arabia saw this as a great opportunity to develop the specialty on a national scale. The process of the development of last phase interventions was a long and meticulous one and has been very successful. This paper attempts to describe the findings from this meticulous developmental process of transformation.

Health is a universal right of all populations. A holistic concept of health includes balance, energy systems, and mind-body integration from birth to the end of life. A healthy population expresses positive and pleasurable behaviour, regardless of their health continuum status [2]. In general, the average lifespan of the Middle East population is between 62 and 70 years for the Saudi population [2]. However, an alarming health issue is the exponentially growing number of cancer diagnoses. The cancer morbidity and mortality in Saudi Arabia appear to be similar to that in Western countries because of a comparable pattern of diseases, such as cancer, wherein 1:3 people has cancer and 1:4 dies of it; 22% of all deaths are cancer-related, as in the United States [3]. Cancer was widely known as a disease of old age; however, because of the dynamic changes in society, this has become a disease of both genders and all ages. In 2008, the Saudi Cancer Registry registered overall cancer cases reaching 11,946 [4], and 13,300 were treated across the Kingdom [5]. In 2030, the rate of cancer cases in Saudi Arabia is expected to surge five to 10 times the current statistics because of continuous changes in the population demographics, particularly with middle-aged and older people [6].

In the past, palliative care was not part of the services provided by interdisciplinary specialties in Saudi Arabia, and there was no integration of palliative care programs within the healthcare system [2]. Subsequently, palliative care programs were assimilated within the systems where they focused on hospital organizations but did not include it as a national standard end of life quality indicator [4]. In 1992, the King Faisal Specialist Hospital and Research Center in Saudi Arabia established a palliative care facility which gradually expanded across the Kingdom [2]. Most undergraduate medical schools were integrating palliative care training in the curriculum and introduced postgraduate training programs in palliative care. In addition, the multidisciplinary team underwent training in palliative care and opened spiritual care for palliative patients. At present, there are 12 existing palliative care centers scattered across the Kingdom and six of them have obtained accreditation from the Saudi Commission for Health Specialties (SCFHS).

Although there are significant achievements in the palliative care specialty in Saudi Arabia, the general population’s knowledge of the end of life cancer care accompanying palliation is limited as the focus is on a cure. End of life care is the care given to people who are near the end of life and have stopped treatment to cure or control their disease. Islamic culture adopts the end of life care and legally governs Saudi Arabia based on the Holy Qur'an [7]. The goal of the end of life care is to control pain and other symptoms so the patient can be as comfortable as possible while maintaining emotional, social, and spiritual support for patients and their families. Apart from a vast number of cancer patients, a good number of patients with chronic but non-cancerous diseases also have equal rights to receive such holistic end of life care.

In order to make the end of life care in Saudi Arabia more patient-centered, a new end of life model of care, “Support me in the last phase of my life,” was designed based on systems of care in 2017. In this new model, patients will be asked six questions related to care from birth up to supporting their loved ones when they die. The questions are: 

1) How will the system support me to keep well?

2) How will the system support me when I have an urgent problem?

3) How will the system support me to have a great outcome for my planned procedure?

4) How will the system support me to safely deliver a healthy baby?

5) How will the system support me with my chronic conditions?

6) How will the system provide me with compassionate care during the last phase of my life?

Meanwhile, to promote a continuous integrated network across the regional clusters in the Kingdom, the care system will work as a service layer to efficiently deliver health care services, promoting prevention rather than a curative approach. The first layer is the individuals and their families keeping themselves well and taking care of their own health. The second layer underlines the role of healthy communities in leading their people towards a healthy lifestyle, correct information, and access to different wellness facilities. The third layer is the role of virtual care as a technological source of health information and services. Beyond the third layer is primary care, secondary care, and tertiary and quaternary care, respectively.

The aim of this study was to identify the positive aspects of end of life care pathways and barriers to the delivery of quality care.

## Materials and methods

A descriptive analysis of the process involved in the development of the last phase is a model of care initiative based on the output reports from the Care Design Group (CDG1-3). An environmental scan of workshop participation inviting 61 individuals, including senior healthcare professionals, policymakers in cancer care, and patients, for developing end of life care pathways was conducted. Following this, 2,500 clinicians (including researchers and policymakers) took part in the online meetings, discussions, and surveys over a two month period. The responses were compiled to form a long list of system-related issues. A summary of this survey will be described.

## Results

Environmental survey of life care workshop

**Figure 1:**
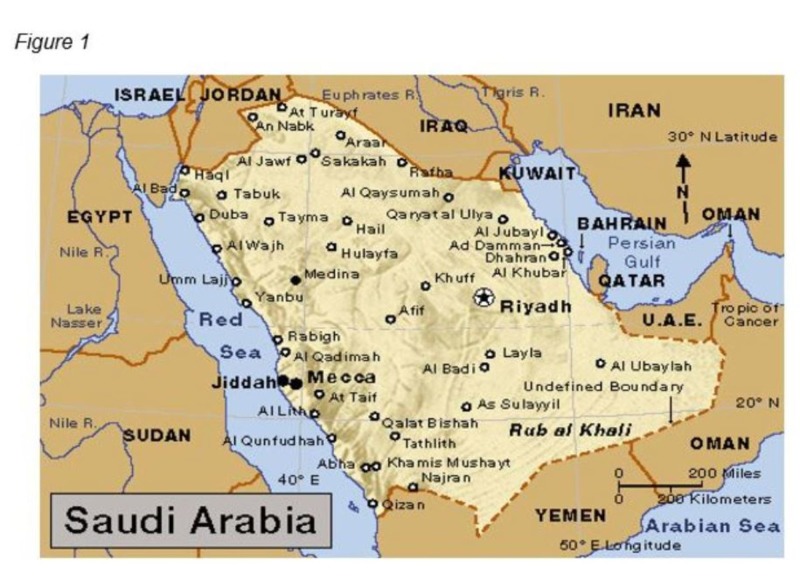
Average Distances from Different Parts of Saudi Arabia

Sixty-one senior health care professionals, policymakers in cancer care, and patients were invited. Forty-six of them sent an email confirmation and participated in the workshop. The overall response rate was 75.4%. Regional attendees were higher in Riyadh (46%), followed by the Eastern province (11%). However, there were no workshop attendees from Al Baha, AlAhsa’a, Gurayat, Hafer Albatin, AlHa'il, Najran, and Qunfudah due to the distance from the workshop venue. Please see Figure 1 for the average distances from different parts of Saudi Arabia. Meanwhile, the MOH (56%) had the highest number of attendees, followed by the medical cities (15%). We had three patients out of the invitees attending these workshops. Health care in Saudi Arabia can be classified as a national health care system in which the government provides health care services through a number of government agencies. In the meantime, there is a growing role and increased participation from the private sector in the provision of health care services. The MOH is the major government agency entrusted with the provision of preventive, curative, and rehabilitative health care for the Kingdom’s population. The Ministry provides primary health care (PHC) services through a network of health care centers (comprising 1,925 centers) throughout the Kingdom. In what could be the beginning of an international medical renaissance, medical cities could change the way medical education, research, and development is conducted, taking it from the public to private to corporate domain. Once individual international hospitals are now combined, each to provide specific areas of treatment, shared expenses, and lower costs to the patient. Please see Table 1 for a list of Medical Cities.

**Table 1:** List of Medical Cities

Medical Cities	
1	Prince Mohammed Ibn Abdulaziz Ibn Abdulrahman Medical City
2	King Fahad Medical City
3	King Saud Medical City
4	King Saud University Medical City
5	King Abdullah Medical City - Riyadh
6	King Faisal Medical City - Aseer
7	Prince Sultan Military Medical City
8	King Abdulaziz Medical City
9	King Khalid Medical City
10	King Abdullah Specialized Children’s Hospital
11	King Abdulaziz Hospital
12	Imam Abdulrahman Al Faisal Hospital
13	King Abdulaziz Medical City - Jeddah
14	King Faisal Specialist Hospital and Research Center

King Saud Medical City organized this workshop to help in the redesigning and development of a model of care (MoC) for patients with life-shortening illnesses in Saudi Arabia. This was conducted on January 19, 2017, and started from 8 am until 4 pm.

The summary of geographic and organizational distributions are shown in Table 2.

**Table 2:** Summary of Workshop Attendees by Region and Organization

Regions	No. of attendees n = 46	Organizations	No. of attendees n = 46
	f (%)		f (%)
Aljouf	1 (2)	Ministry of Health	26 (56)
Al-Madinah	2 (4)	Medical City	7 (15)
Asir	2 (4)	Other government	6 (13)
Bisha	1 (2)	Private	4 (9)
Eastern	5 (11)	Patients	3 (6)
Jeddah	5 (11)		
Jizan	1 (2)		
Makkah	3 (7)		
Northern	1 (2)		
Qassim	3 (7)		
Riyadh	21 (46)		
Tabuk	1 (2)		

Out of these pathways, extraction of the best solutions was accomplished by the voting of the 46 experts according to current priorities, then again underwent successive voting until finally the top five immediate priority pathways were identified, namely: 1) bereavement, 2) current and future treatment plans, 3) monitoring, 4) individual and career support, and 5) early identification of terminal phase.

Forty-six multidisciplinary end of life healthcare senior professionals, policymakers working in the cancer sector, and patients were involved in the development process. After a series of workshops, virtual meetings, and discussions, there were seven end of life system of care pathways under “Support me in the last phase of my life.”

Online survey and discussions

**Figure 2:**
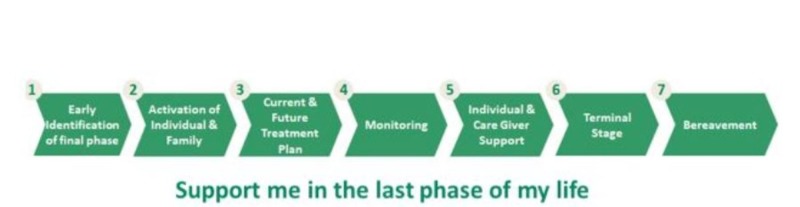
Seven Key Dimensional Pathways

Nearly 2,500 clinicians took part in the online meetings, discussions, and surveys over a two month period. The responses were compiled to form a long list of system-related issues; after review, it was decided to create a summary focused on key priority issues which concluded with the seven key dimensional pathways (Figure 2).

Among the list of solutions under the seven pathway components, psychosocial and financial support (48%) under bereavement got the highest priority, followed by the formation of a specific treatment plan (39%), developing mobile applications and hotlines (35%), data collection and update (35%), and the development of a clear policy and procedures (33%) for early identification of the end of life phase. All of the top three solutions of every component were essential; however, a few solutions listed have to undergo further review as they required workshop/classes for caregiving needs, resources to build a place for terminal stage patients, and building a nationwide network for an electronic bereavement support system. Frequencies and percentages were based on 46 participants. Table 3 represents the top three solutions for "Support me in the last phase of my life pathway."

**Table 3:** Support Me in the Last Phase of My Life - Top Three Solutions of Each Pathway Component App: application; IT: information technology

Step	End of life care pathways	Solutions	f (%)
1	Early identification of the final phase	Develop a clear policy and procedures	15 (33)
		Increase awareness of healthcare providers and community in end of life care	12 (26)
		Set a well-established national referral palliative care form	6 (13)
2	Activation of individual and family	Home health care service implementation	14 (30)
		Family meeting	10 (22)
		IT application (hotline and app)	9 (19)
3	Current and future treatment plans	A multidisciplinary team approach to form a specific treatment plan	18 (39)
		A proper utilization of home care services	10 (22)
		Patient/family center for a model of care	4 (9)
4	Monitoring	Data collection and update	16 (35)
		Logistic support	10 (22)
		Using the technology (mobile app and webpage)	3 (6)
5	Individual and caregiver support	Develop mobile app and hotline	16 (35)
		Good healthy environment	10 (22)
		Teaching workshops/classes for caregiving needs	5 (11)
6	Terminal stage	Psychosocial support	11 (24)
		Protocols review	8 (17)
		Setting/place	4 (9)
7	Bereavement	Psychosocial and financial support	22 (48)
		Special training for healthcare providers	7 (15)
		Electronic bereavement support system	5 (11)

A detailed strengths, weaknesses, opportunities, and threats (SWOT) analysis of the responses was done. The details of the SWOT analysis and the final model of care (MoC), last phase interventions, and pathway will be published as a separate report.

## Discussion

This is the first published report discussing an integrated end of life care system development in the Middle East. It is hoped that the results of this report will serve to stimulate further discussion about the use of the typology for raising awareness of the need for integrating palliative care into the existing healthcare systems. The increased interest in access to palliative care as a human right provides further justification for examining the relationship between palliative care services and their accessibility to the populations of individual countries [8]. The problems of palliative care in Saudi Arabia can be summarized as follows: 1) the strong emphasis on "cure," even when this is no longer possible; 2) the lack of physicians with an interest in palliative care; 3) the fact that patients generally are not told their diagnosis of cancer and have no idea of their prognosis. Saudi Society is paternalistic. Women and the elderly are usually "protected" from receiving bad news. While some of the patients, especially the elderly, clearly waive off their right to know and want the discussions to be done with their appointed power of attorney, there still remains a significant number of patients who are not given the entire details of their diagnosis and prognosis due to well-intended collusion; 4) the lack of a family health service, integrated with secondary and tertiary care, to provide continuity of care in the community; 5) the lack of adequate methods of pain relief; and 6) the unwillingness to discuss issues of death and dying [9]. Many other developed countries have identical problems. The Kingdom, however, has the advantage of a centrally organized, government-supported health service to help plan and implement such services on a national level [10]. Although there have been great advancements in cancer treatment, there comes a stage where more treatment might cause more harm than good or the cancer is simply non-responsive to treatment. While our ability to improve survival is still limited, the ability to control symptoms and improve quality of life has continually improved. Dr. Neill McDonald, a Canadian expert, recently wrote that he considers palliative care to be one of the four major advances in the clinical management of cancer patients in the last decade [11]. He lists these as adjuvant chemotherapy of some cancers, the control of nausea and vomiting, palliative care, and progress in bone marrow transplantation. In the final analysis, palliative care is simply good medicine, and there is no one group that has a unique hold on the humane management of pain and psychosocial issues. The philosophy of palliative care is best integrated within a health system where it can permeate and influence the care of cancer (and other) patients at all stages of their illness. Developing palliative care programs throughout the Kingdom now would speed up that process [12].

## Conclusions

The project “Support me in the last phase of my life” was a timely approach to address the country’s demand for palliative care. Hospital and community’s mutual coordination was the best strategy to improve a cancer patient’s quality of life, empower cooperation among patients and careers, cost-saving hospice care, up-to-date learning, and increase job opportunities. Although changes have been affecting the public/private/community sectors, the Ministry of Health’s objective on Vision 2030 is for the good of all. Meanwhile, the seven care pathways under this project were essential portals towards national palliative care development. However, challenges in achieving these critical points include needing the support of all involved stakeholders on national guidelines for prescribing, referral and treatment, developing home palliative care services, educational programs for target groups (workshop and social media), a national data warehouse, creating a mobile app and hotlines, developing processes and structures for family meetings, and psycho/social/spiritual and financial support. As our population ages, there will be a growing need for palliative care programs. The demand for improved home and community programs will increase as many people in need of palliative care will prefer to stay at home or reside in long-term care settings. The progressive shift towards integrating palliative care, alongside a standard care for patients with advanced and severe illnesses, will also compound the need for improved access to, and equity in, palliative care programs.
